# Myocardial ischaemia following COVID-19: a cardiovascular magnetic resonance study

**DOI:** 10.1007/s10554-024-03304-7

**Published:** 2024-12-30

**Authors:** J. Ranjit Arnold, Jian L. Yeo, Charley A. Budgeon, Simran Shergill, Rachel England, Hunain Shiwani, Jessica Artico, James C. Moon, Miroslawa Gorecka, Giles Roditi, Andrew Morrow, Kenneth Mangion, Mayooran Shanmuganathan, Christopher A. Miller, Amedeo Chiribiri, Mohammed Alzahir, Sara Ramirez, Andrew Lin, Peter P. Swoboda, Adam K. McDiarmid, Robert Sykes, Trisha Singh, Chiara Bucciarelli-Ducci, Dana Dawson, Marianna Fontana, Charlotte Manisty, Thomas A. Treibel, Eylem Levelt, Robin Young, Alex McConnachie, Stefan Neubauer, Stefan K. Piechnik, Rhodri H. Davies, Vanessa M. Ferreira, Marc R. Dweck, Colin Berry, Oxford Acute Myocardial Infarction OxAMI Study Investigators, COVID-HEART investigators, Gerry P. McCann, John P. Greenwood

**Affiliations:** 1 University of Leicester and The NIHR Leicester Biomedical Research Centre, Glenfield Hospital, Leicester, UK; 2 Cardiovascular Epidemiology Research Centre, School of Population and Global Health, University of Western Australia, Perth, Australia; 3 Institute of Cardiovascular Science, University College London, London, UK; 4 Institute of Cardiovascular and Metabolic Medicine, University of Leeds, and Leeds Teaching Hospitals NHS Trust, Leeds, UK; 5 School of Cardiovascular and Metabolic Health, University of Glasgow, Glasgow, UK; 6 Institute of Cardiovascular and Medical Sciences and British Heart Foundation Glasgow Cardiovascular Research Centre, University of Glasgow, Glasgow, UK; 7 Division of Cardiovascular Medicine, Radcliffe Department of Medicine, Oxford Centre for Clinical Magnetic Resonance Research, Oxford, UK; 8 British Heart Foundation Centre of Research Excellence, Oxford, UK; 9 Oxford NIHR Biomedical Research Centre, University of Oxford, Oxford, UK; 10 Division of Cardiovascular Sciences, School of Medical Sciences, Faculty of Biology, Medicine and Health, University of Manchester, Manchester, UK; 11 School of Biomedical Engineering and Imaging Sciences, King’s College London, BHF Centre of Excellence and The NIHR Biomedical Research Centre at Guy’s and St. Thomas’ NHS Foundation Trust, The Rayne Institute, St. Thomas’ Hospital, London, UK; 12 Adult Congenital and Paediatric Heart Unit, Freeman Hospital, Newcastle Hospitals NHS Foundation Trust, Newcastle Upon Tyne, UK; 13 University of Edinburgh and British Heart Foundation Centre for Cardiovascular Science, Edinburgh, UK; 14 Royal Brompton and Harefield Hospitals, London, UK; 15 Guys’ and St Thomas NHS Trust, London, UK; 16 Bristol Heart Institute, University Hospitals Bristol and Weston NHS Trust, Bristol, UK; 17 Department of Cardiology, Aberdeen Cardiovascular and Diabetes Centre, Aberdeen Royal Infirmary and University of Aberdeen, Aberdeen, UK; 18 Division of Medicine, Royal Free Hospital, University College London, London, UK; 19 Robertson Centre for Biostatistics, Institute of Health and Wellbeing, University of Glasgow, Glasgow, UK; 20 Baker Heart and Diabetes Institute, Melbourne, Australia

**Keywords:** Cardiovascular diseases, Coronavirus, COVID-19, Magnetic resonance imaging, Myocardial ischaemia

## Abstract

The pathophysiology of myocardial injury following COVID-19 remains uncertain. COVID-HEART was a prospective, multicentre study utilising cardiovascular magnetic resonance (CMR) to characterise COVID-related myocardial injury. In this pre-specified analysis, the objectives were to examine (1) the frequency of myocardial ischaemia following COVID-19, and (2) the association between ischaemia and myocardial injury. We studied 59 patients hospitalised with COVID-19 and elevated serum troponin (COVID + /troponin + , age 61 ± 11 years) and 37 control subjects without COVID-19 or elevated troponin and similar by age and cardiovascular comorbidities (COVID −/comorbidity + , 64 ± 10 years). Subjects underwent multi-parametric CMR (comprising assessment of ventricular volumes, stress perfusion, T1/T2 mapping and scar). The primary endpoint was the frequency of inducible myocardial ischaemia. Inducible ischaemia was evident in 11 (19%) COVID + /troponin + patients and in 8 (22%) control subjects (*p* = 0.72). In COVID + /troponin + patients with ischaemia, epicardial coronary disease pattern ischaemia was present in eight patients and microvascular disease pattern, in three patients. There was no significant difference in the frequency of inducible ischaemia in COVID + /troponin + patients with previous myocardial infarction and/or revascularisation compared to those without (2/12 [17%] vs. 9/47 [19%] respectively, *p* = 0.84), or in those with and without scar (7/27 [26%] vs. 4/32 [13%] respectively, *p* = 0.19). Myocardial ischaemia was present in ~ 20% of patients recently hospitalised with COVID-19 and with elevated cardiac troponin, but this was not different to matched comorbid controls. This finding coupled with the lack of an association between ischaemia and myocardial scar suggests that coronary artery abnormalities are unlikely to be the predominant mechanism underlying COVID-19 induced myocardial injury.

**Graphical abstract:**

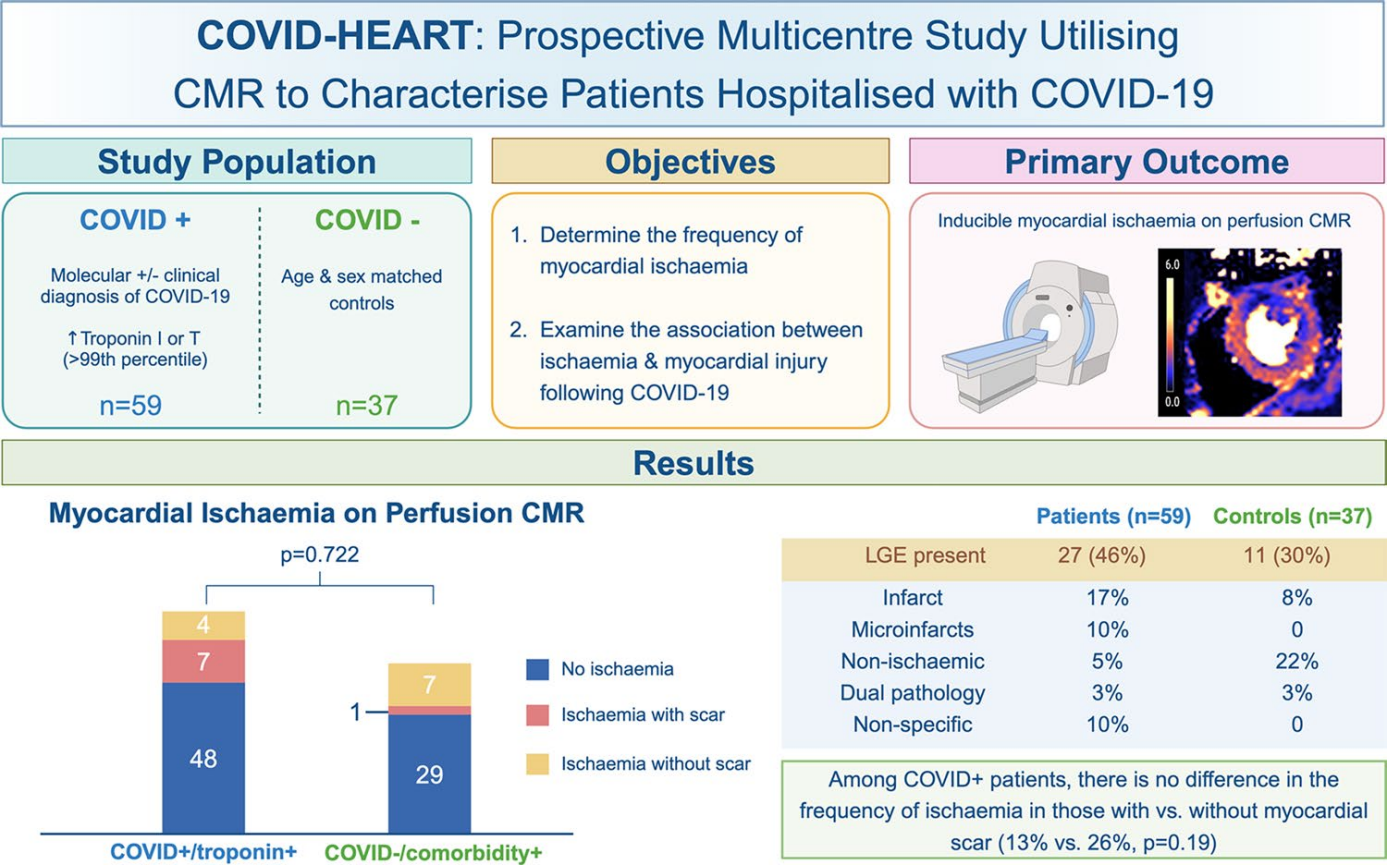

**Supplementary Information:**

The online version contains supplementary material available at 10.1007/s10554-024-03304-7.

## Introduction

The novel disease (COVID-19) caused by the SARS-CoV-2 virus was the most severe pandemic since the 1918 influenza pandemic, with critical health and economic consequences internationally. To date, over 770 million cases and 7 million deaths have been recorded [1]. Although the virus predominantly affects the respiratory system, a broad spectrum of disease phenotypes have been observed, with multi-organ involvement being associated with worse clinical outcome [2, 3]. Cardiac injury, as evidenced by elevated serum troponin, has an incidence of 15–20% in patients hospitalised with COVID-19, and is associated with a higher rate of mortality. The presence of pre-existing cardiovascular comorbidities and coronary risk factors is associated with higher risk of hospitalisation and mortality, potentially implicating atherothrombotic disease mechanisms [4]. Acute-phase, severe COVID-19 disease is characterised by vascular endothelial damage and hypercoagulability, with a high incidence of thrombotic events in multiple organ systems [5–8]. Hence, vascular disease mechanisms may also underlie the acute cardiac injury. Other potential injury mechanisms include direct viral cytopathic damage (leading to myopericarditis) or type II myocardial infarction (due to oxygen supply–demand mismatch). Currently, there remains uncertainty regarding the relative importance of ischaemic and non-ischaemic mechanisms of myocardial injury in COVID-19.

Few studies have explored the potential mechanistic link between ischaemia and myocardial injury: previous studies have primarily comprised small or single-centre, retrospective analyses, involving convenience samples of patients presenting with chest pain/dyspnoea or those referred clinically for perfusion assessment [9–13]. By contrast, COVID-HEART was a prospective, multicentre study utilising cardiovascular magnetic resonance (CMR) to characterise the prevalence and mechanisms of myocardial injury in patients hospitalised with COVID-19 and with serum troponin elevation [14, 15]. In this pre-specified analysis, we evaluated the findings from stress perfusion CMR: (1) to determine the frequency of myocardial ischaemia following COVID-19, and (2) to explore whether persistent myocardial ischaemia (macrovascular and/or microvascular) may contribute to myocardial injury.

## Methods

Patients were prospectively recruited in an observational cohort study conducted at 25 secondary or tertiary care centres (ISRCTN 58667920) in the United Kingdom. Study methodology and baseline results have been published previously [14, 15]. In brief, the study comprised adult patients hospitalised with COVID-19 (confirmed with molecular and/or clinical diagnosis) and elevated high sensitivity cardiac troponin I or T (greater than the sex-specific 99th centile). Exclusion criteria were: inability or unwillingness to consent, contraindication to CMR, pregnancy or breastfeeding. For the present analysis, data from all participants who underwent stress CMR perfusion imaging were evaluated. A control group comprising age and cardiovascular comorbidity matched subjects without COVID-19 (COVID − /comorbidity +) was also studied (recruited prospectively at five COVID-HEART centres as part of ongoing research studies (e.g. CISCO-19, PREDICT, C-MORE, OxAMI, as previously described) [15–18]. All subjects provided written informed consent prior to participation. The study conforms to the ethical guidelines of the 1975 Declaration of Helsinki and was approved by the UK National Research Ethics Service (20/NW/0292) and data sharing under a UK national control of patient information (COPI) agreement.

### CMR

CMR scans were performed on either a 1.5 or a 3 T magnetic resonance system, for COVID-19 cases, during their index admission or within 28 days of discharge, and for control subjects, as outpatients. The CMR protocol has been previously described [14]. In brief, this comprised anatomic imaging (transaxial stack), functional cine imaging using a steady-state free precession (SSFP) pulse sequence, late gadolinium enhancement (LGE) imaging and if available, myocardial tissue characterisation including T2 mapping, pre- and post-contrast T1 mapping. Additionally, stress/rest perfusion imaging was carried out with the locally available pulse sequence (pixel-wise perfusion mapping where available [19]) following administration of 0.05 mmol/kg of gadolinium-based contrast agent (GBCA) injected at 4 mL/s followed by a 20 mL flush injected at 4 mL/s [19]. For adenosine stress perfusion imaging, 140–210 mcg/kg/min adenosine (depending on hemodynamic and symptomatic response) was infused for at least three minutes.

### CMR image analysis

CMR datasets were analysed blinded to disease status within a disseminated core laboratory (Leicester: perfusion; Barts: structure/function/LGE; Oxford: T1/T2 mapping; Glasgow: extracardiac anatomy). LGE phase-sensitive inversion recovery images were analysed by a consensus of experienced independent observers and quantified using a semi-automated signal intensity analysis (5-standard deviation). Details on LGE analysis, volumetric assessment and tissue characterisation have been previously reported [14, 15].

CMR perfusion images were analysed using cvi42 v5.12.1 (Circle Cardiovascular Imaging, Calgary, Canada). Perfusion defects were first evaluated semi-quantitatively by two observers (JRA/GPM) acting in consensus, as previously described [14]. For each perfusion scan, image quality was rated on a 4-point scale (3—excellent, 2—good, 1—moderate, 0—unanalysable). The presence of artefact was also recorded. Perfusion scans were evaluated side by side with LGE images from the closest corresponding short-axis image positions. The presence of scar in these slice positions was recorded as present or absent, and categorised as subendocardial or transmural (using the 16-segment AHA model). For the independent, patient-level assessment of scar, all slice positions were evaluated. For each perfusion scan, the presence of perfusion defects was recorded using the 16-segment AHA model, with subdivision of each segment into endocardial/epicardial layers (0—absent perfusion defect, 1—subendocardial, 2—transmural) and ischaemia burden quantified as previously described [20].

Quantitative perfusion (where available) was outputted for each of the 16 segments; stress myocardial blood flow (sMBF), resting MBF (rMBF), and myocardial perfusion reserve (MPR—defined as the ratio of sMBF to rMBF). Quantitative flow assessment, if available, was evaluated to provide an integrated perfusion assessment incorporating both qualitative and quantitative assessments. Microvascular dysfunction was defined as MPR < 2.0 with no regional defects suggestive of epicardial coronary disease [21].

### Statistical analysis

Continuous data are expressed as mean (± standard deviation) or median [interquartile range] and groups were compared with independent sample *t*-tests or Mann–Whitney tests respectively. Categorical data are presented as number (percentage) of patients, and comparisons were performed with Chi-square or Fisher’s exact tests as appropriate. Linear mixed effects models were used to compare patients to control subjects based on the segmental analysis of the CMR images. Subsequent analyses adjusted for potential confounders including scar presence, age, hypertension, smoking status, diabetes and previous beta-blocker use. Statistical analyses were performed using R for statistical computing [22]. *P* < 0.05 was considered statistically significant.

## Results

**Fig. 1:**
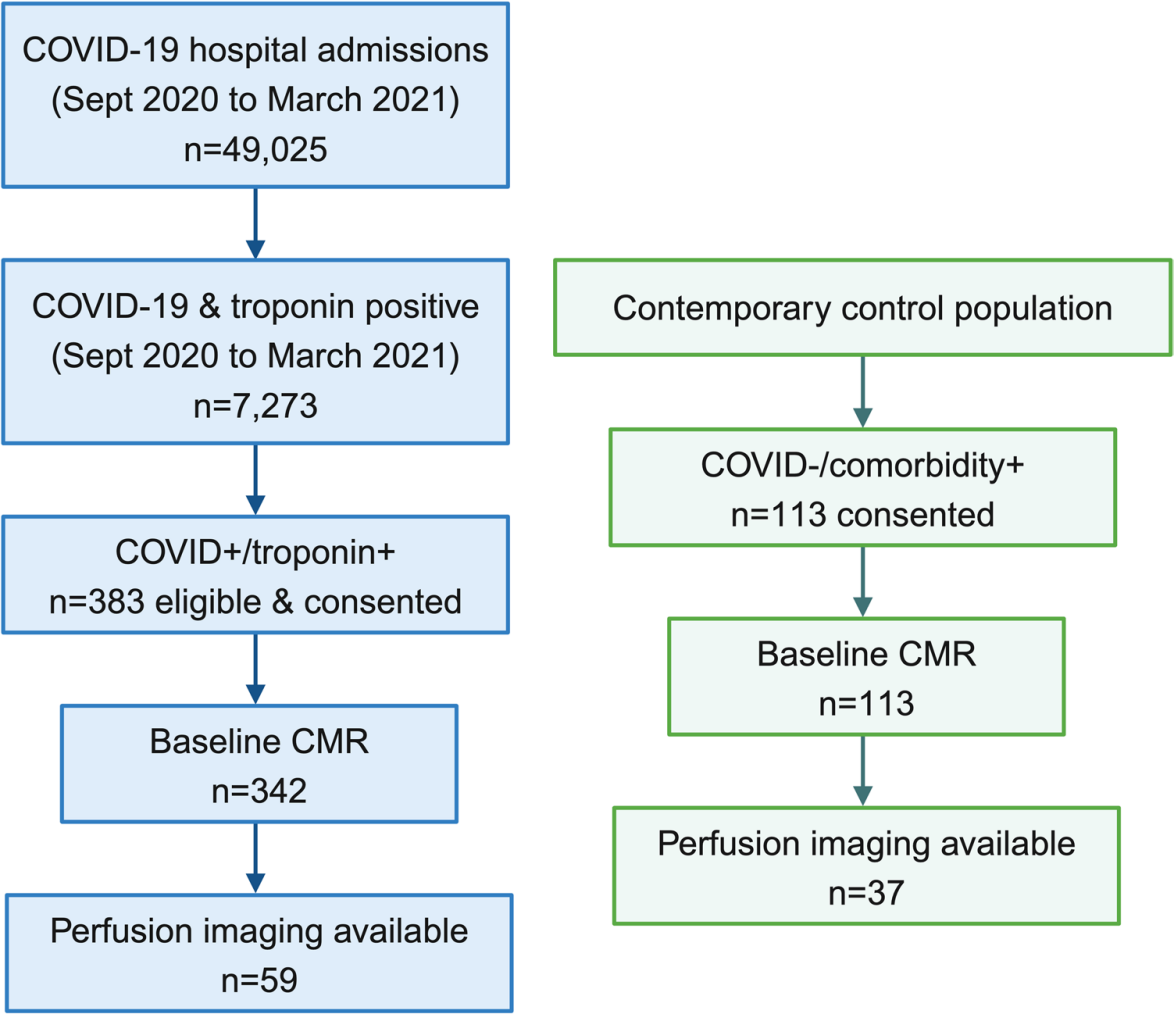
Study flow diagram. Flow diagram of participant recruitment

The flow diagram of participant recruitment is shown in Fig. 1. CMR stress perfusion imaging was performed in 59 of 342 (17%) COVID + /troponin + patients and 37 of 113 (33%) COVID − /comorbidity + control subjects. No patient in this analysis underwent revascularisation during their index admission prior to CMR stress perfusion assessment.

COVID + /troponin + patients who underwent stress CMR (*n* = 59) were generally well matched with those patients who did not undergo perfusion imaging for most baseline and clinical characteristics (*n* = 283, Supplementary Tables 1 and 2). There was a higher preponderance of males (83 vs. 69%, *p* = 0.025) and minority ethnicity in the group who underwent perfusion compared to those who did not undergo perfusion assessment. Additionally, in those who underwent perfusion imaging, there was more frequent use of beta-blockers and angiotensin converting enzyme inhibitors/angiotensin receptor antagonists.

### Characteristics of the subjects undergoing CMR perfusion imaging

Table 1 includes baseline demographics, clinical and imaging characteristics for all subjects who underwent CMR perfusion imaging. COVID + /troponin + patients (*n* = 59) and COVID-/comorbidity + subjects (*n* = 37) were of similar age (mean 61 ± 11 vs. 64 ± 10 years, *p* = 0.219) with a similar distribution between males and females. The prevalence of previous acute coronary syndrome, revascularisation, heart failure, and hypertension was comparable between the groups. In contrast, diabetes was more prevalent in the control group than in COVID + /troponin + patients (43 vs 22%; *p* = 0.028). Medication use was similar between the two groups. In COVID + /troponin + patients who underwent CMR perfusion imaging, the median length of hospital stay was seven days, and subjects were scanned a median of 21 days following hospital discharge.

**Table 1:** Clinical characteristics of COVID + /troponin + patients and COVID−/comorbidity + control subjects undergoing perfusion assessment

	Patients	Controls	*P*-value
	(*n* = 59)	(*n* = 37)	
Age (years)	60.8 ± 11.4	63.6 ± 9.7	0.219
Sex (Male)	49 (83.1%)	29 (78.4%)	0.568
Body mass index (kg/m^2^)	29.9 ± 6.3	29.1 ± 8.8	0.532
Body surface area (m^2^)	–	1.99 ± 0.22	–
Charlson Score	2.64 ± 1.89	–	–
Haemoglobin (g/L)	137 ± 20.7	150.1 ± 13.5	0.002
eGFR (ml/min/1.73m^2^)	63.2 ± 22.6	N/A	–
Time of CMR post-hospital discharge (days)	21 [15-27]	–	–
Hospital length of stay (days)	7 [4-13]	–	–
Previous MI/ACS*	8 (13.6%)	4/35 (11.4%)	1
Previous revascularisation*	9 (15.3%)	2/35 (5.7%)	0.202
Hypertension	27 (45.8%)	17 (45.9%)	0.986
Heart failure	6 (10.2%)	7/35 (20%)	0.222
Smoking status			0.003
Current	0 (0%)	7 (18.9%)	
Former	18 (30.5%)	9 (24.3%)	
Never	41 (69.5%)	21 (56.8%)	
Diabetes (any)	13 (22.0%)	16 (43.2%)	0.028
Aspirin/Clopidogrel/Ticagrelor	15 (25.4%)	N/A	–
Statin	27 (45.8%)	20 (54.1%)	0.429
Beta-blocker	20 (33.9%)	12 (32.4%)	0.882
ACE-I or ARB	28 (47.5%)	17 (45.9%)	0.885
Anticoagulant	4 (6.8%)	N/A	–

Data presented are mean ± SD, median [Q1–Q3], or n (%). Abbreviations: *ACE-I* angiotensin converting enzyme inhibitor; *ARB* angiotensin receptor blocker; *ACS* acute coronary syndrome; *CMR* cardiovascular magnetic resonance imaging; *MI* myocardial infarction, N/A all data missing for controls. Denominator is 37 for controls unless otherwise stated. *Denotes prior to index COVID-19 admission

All CMR perfusion images were of sufficient quality, and no images were excluded from analysis (83% graded excellent quality and 11% good). Regarding CMR indices, compared with COVID − /comorbidity + subjects, COVID + /troponin + patients had similar ejection fraction (62 ± 1 vs 63 ± 1%, *p* = 0.704) but higher LV mass index (62.5 ± 18.1 vs. 52.9 ± 10.1 g/m^2^, *p* = 0.001) and more concentric LV remodelling (mass/volume 0.78 ± 0.21 vs. 0.69 ± 0.11 g/ml, *p* = 0.009). There was a trend towards more scar in the COVID + /troponin + patients (46 vs 30%) but this did not reach statistical significance (*p* = 0.118) (Table 2).

**Table 2:** Imaging characteristics of COVID + /troponin + patients and COVID− /comorbidity + control subjects undergoing perfusion assessment

	Patients	Controls	*P*-value
	(*n* = 59)	(*n* = 37)	
Left ventricular end-diastolic volume index (ml/m^2^)	83.4 ± 27.6	78.6 ± 19.5	0.329
Left ventricular end-systolic volume index (ml/m^2^)	33.4 ± 27.8	31.2 ± 18.2	0.641
Left ventricular mass index (g/m^2^)	62.5 ± 18.1	52.9 ± 10.1	0.001
Left ventricular ejection fraction (%)	62.9 ± 13.6	61.9 ± 13.4	0.704
LVMi/LVEDVi (g/ml)	0.78 ± 0.21	0.69 ± 0.11	0.009
LGE present	27 (45.8%)	11 (29.7%)	0.118
Infarct	10 (17.0%)	3 (8.1%)	
Non-ischaemic	3 (5.1%)	7 (21.6%)	
Dual pathology	2 (3.3%)	1 (2.7%)	
Nonspecific	6 (10.2%)	0	
Microinfarcts	6 (10.2%)	0	
Inducible ischaemia present	11 (18.6%)	8 (21.6%)	0.722
Ischaemic burden (%)	3.81 ± 9.73	6.67 ± 14.81	0.302
Myocardial perfusion reserve	2.63 ± 0.85	2.94 ± 1.30	0.249
Myocardial perfusion reserve < 2	12/47 (25.5%)	5/29 (17.4%)	0.572

Data presented are mean ± SD, median [Q1–Q3], or *n* (%). Abbreviations: *LGE* late gadolinium enhancement; *LVMi* left ventricular end-diastolic mass indexed to body surface area; *LVEDVi* left ventricular end-diastolic volume indexed to body surface area

### CMR perfusion data

**Fig. 2:**
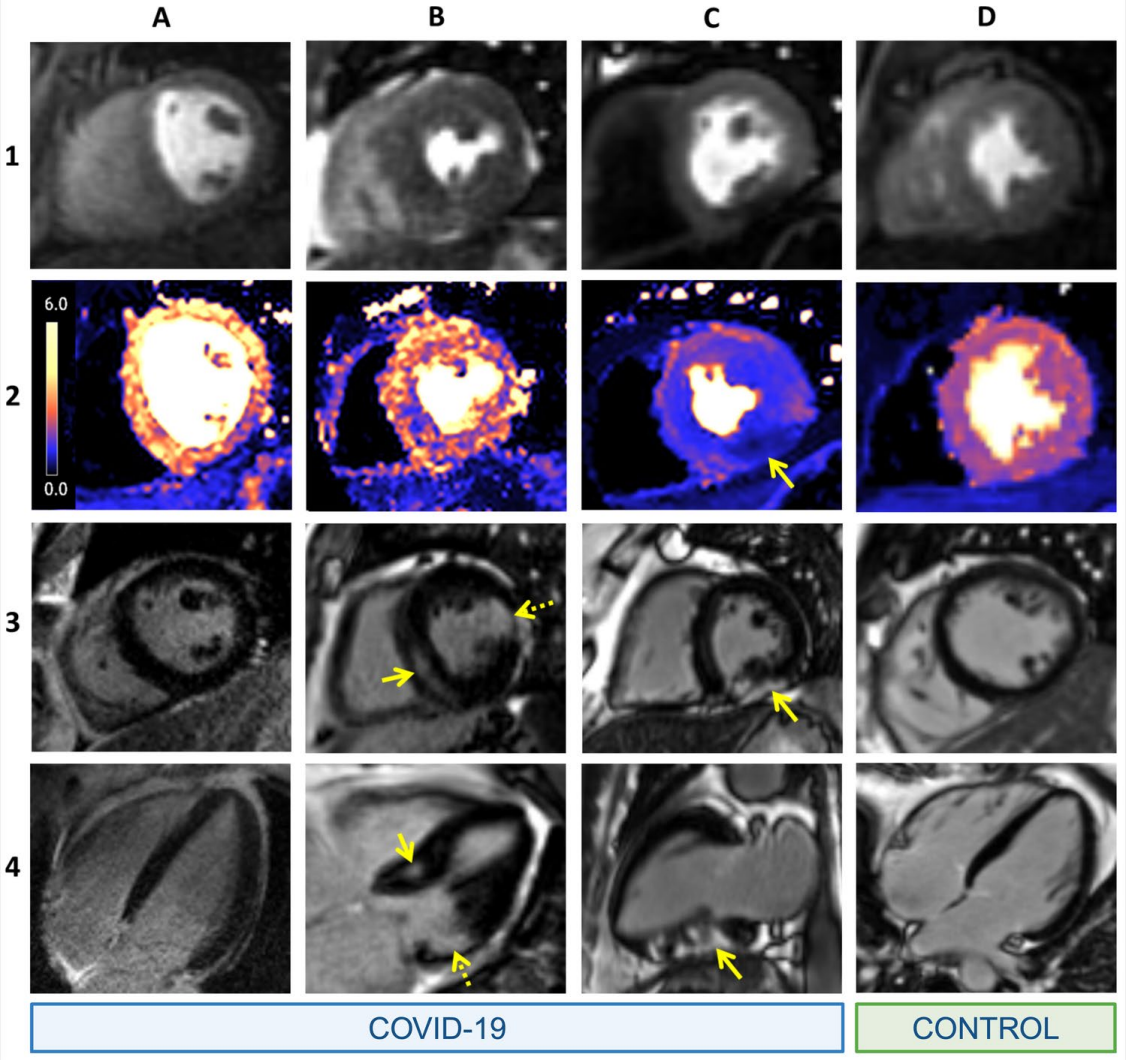
Selected patient examples. CMR images displaying: (**1**) stress first-pass perfusion, (**2**) pixel-wise stress perfusion map, (**3**) short-axis and (**4**) long-axis late gadolinium enhancement (LGE) of COVID + /troponin + patients (**a to d**) and a COVID − /comorbidity + subject (**e**). **a** 48 year old male with no regional hypoperfusion, global myocardial perfusion reserve (MPR) of 4.0 and no LGE. **b** 72 year old male with no regional hypoperfusion. Mid-wall LGE in the basal inferoseptum (solid arrow) and subendocardial infarct in the basal anterolateral wall (dotted arrow). **c** 50 year old male with inferior segment perfusion defect with subepicardial LGE and a microinfarct in this segment (arrows). Global MPR 1.8. **d** 54 year old male control with normal perfusion and no LGE. Global MPR 4.1

Inducible ischaemia was evident in 11 (19%) of COVID + /troponin + patients compared to 8 (22%) of COVID − /comorbidity + subjects (*p* = 0.722). In those in whom ischaemia was present, the average left ventricular ischaemia burden was 20% in COVID + /troponin + patients compared to 31% in control subjects (*p* = 0.146, Fig. 2 for patient examples). In COVID + /troponin + patients with ischaemia (*n* = 11), epicardial coronary disease pattern ischaemia was present in eight patients and microvascular disease pattern, in three. In COVID + /troponin + patients, there was no significant difference in the frequency of inducible ischaemia in those with and without scar, as determined by LGE (4/32 [13%] vs. 7/27 [26%], *p* = 0.187). In the seven COVID + /troponin + patients with both scar and inducible ischaemia, scar pattern was classified as infarction (*n* = 2), microinfarction (*n* = 2), dual pathology (*n* = 1) and non-specific (*n* = 2). The baseline and peak troponin elevation amongst COVID + /troponin + patients was 1.86 times upper limit of normal [IQR 1.27–3.93] and 1.98 [1.33–6.68] respectively, with no significant difference in the degree of troponin elevation in those with and without ischaemia (2.82 times upper limit of normal [1.98–45.56] and 1.82 [1.29–5.19], *p* = 0.110). There was no significant difference in the frequency of inducible ischaemia in COVID + /troponin + patients with a previous history of infarction and/or revascularisation versus those without (2/12 [17%] vs. 9/47 [19%], respectively, *p* = 0.84).

Quantitative perfusion imaging was available in 47 COVID + /troponin + patients and 29 COVID − /comorbidity + subjects. In these individuals, mean global MPR was 2.63 ± 0.85 in COVID + /troponin + patients compared to 2.94 ± 1.30 in control subjects (*p* = 0.249). MPR was lower on average in COVID + /troponin + patients with scar than those without (2.29 ± 0.81 vs. 2.93 ± 0.77, p = 0.008). For those subjects in whom quantitative perfusion assessment was available, microvascular dysfunction was present in 26% of COVID + /troponin + patients and 17% of controls (*p* = 0.572).

For segmental analysis, perfusion data were available for a total of 1211 myocardial segments. Following adjustment for age, hypertension, diabetes, smoking status, beta blocker use and the presence of scar, there was no significant difference in segmental MPR between COVID + /troponin + patients and controls (*p* = 0.138).

## Discussion

In this pre-specified analysis, the principal finding is that persistent myocardial ischaemia is infrequent in patients recently hospitalised with COVID-19 and elevated cardiac troponin. This finding coupled with the lack of an association between ischaemia and myocardial scar suggests that epicardial coronary artery disease (CAD) is unlikely to be the predominant mechanism underlying COVID-19 induced myocardial injury. Rather, it is more likely that myocardial injury in COVID-19 is thrombotic or thromboembolic in origin and hence, standard therapies for acute coronary syndromes may not be warranted in the majority of patients with troponin elevation in COVID-19.

Early studies of acute cardiac injury following COVID-19 revealed abnormalities in up to 78% of patients in some series [11, 23, 24]. However, the prevalence of cardiac pathology attributable to COVID-19 may have been overestimated in small and/or single-centre studies which adopted a retrospective approach and lacked appropriate contemporaneous matched controls. In COVID-HEART, a multicentre, prospective study with contemporaneous matched controls, cardiac abnormalities were observed in 61% of COVID + /troponin + patients, twice the prevalence of that in either COVID + /troponin− subjects (36%) or COVID − /comorbidity + controls (31%) [15]. Infarction and microinfarction were more prevalent (respectively, 13 vs. 2 vs. 7%, and 9 vs. 0 vs. 1%, *p* < 0.01), but non-ischaemic scar was comparable (13 vs. 5 vs. 14% *p* = N.S.). Several explanations are plausible, including a higher prevalence of pre-morbid coronary heart disease in individuals with myocardial injury complicating COVID-19 and second, the vascular pathophysiology of acute COVID-19, including macro- and microangiopathic thrombosis contributing to ischaemic myocardial injury following COVID-19 [25].

Acute cardiac injury in COVID-19 is thought to involve a complex interplay of factors, including direct viral cytopathic effects, immune response dysregulation and dysfunction of the renin–angiotensin–aldosterone system (RAAS) [26, 27]. Although angiotensin-converting enzyme 2 (ACE2) receptor-mediated viral entry and RAAS dysfunction is unique to COVID-19, the damage caused by the excessive activation of the innate immune system and release of pro-inflammatory cytokines is common to sepsis from other aetiologies. These mechanisms may give rise to structural and/or functional abnormalities of the microvasculature, resulting in impaired perfusion and consequent cell damage [28]. In the myocardium, viral interaction with ACE2 receptors expressed on capillary pericytes may increase levels of angiotensin II, a powerful vasoconstrictor which may contribute to vascular injury, as well as augmenting inflammation and thrombogenicity [29–31]. Thus, de novo inflammatory damage to the endothelium and/or augmented inflammatory activity within existing atherosclerotic plaques may ensue. A key feature of COVID-19 is hypercoagulability, mediated by increased platelet activation and degranulation, increased neutrophil extracellular traps and activation of the contact/tissue factor pathways [32, 33]. The resultant microthrombi deposition accounts for the high incidence (25%) of venous thromboembolism and thrombotic events in multiple organ systems [5–8]. Accordingly, elevated levels of D-dimer/fibrinogen are associated with adverse outcomes [34]. Hence, both inflammatory and thrombogenic mechanisms, culminating in obstruction of coronary arteries, may underlie COVID-19-associated damage within the heart. Consistent with this, autopsy studies have confirmed the presence of microthrombi within the coronary microvasculature [35, 36].

Although several CMR studies have investigated myocardial injury, relatively few have evaluated myocardial perfusion following COVID-19. A single-centre retrospective study of patients referred clinically for investigation of chest pain and/or dyspnoea with myocardial perfusion scintigraphy by single-photon emission computed tomography (MPS-SPECT) identified a higher prevalence of ischaemia in patients with previous COVID-19 (23%; 77/329) than in those without (16%; 244/1495) [9]. In a case–control study of 34 post-COVID-19 subjects referred for clinically indicated N13-ammonia myocardial stress perfusion PET (studied a median time of 4.6 months after COVID-19), abnormal myocardial blood flow reserve (< 2) was identified in 44% (15/34) of subjects compared with 12% (12/103) of matched, COVID-19 negative controls (*p* < 0.001) [13]. Reduced MPR was also demonstrated in a small single-centre study evaluating coronary sinus flow using CMR in post-COVID-19 patients (*n* = 22, studied 1–6 months after acute illness) [10]. However, this study was restricted to patients with persistent post-COVID-19 dyspnoea and fatigue, and controls (*n* = 17) were selected retrospectively, with low pretest probability of CAD and were significantly younger (median age 39 years versus 51 years for patients). Another study evaluated 148 patients hospitalised with severe COVID-19 (with troponin elevation, and 32% requiring ventilatory support) [11]. Ischaemia testing with CMR was carried out when a clinical indication was present (*n* = 76, median 68 days following discharge): inducible ischaemia was present in 26% (20/76). In a case–control study of 90 post-COVID patients clinically referred for adenosine stress-perfusion CMR, regional perfusion defects were identified in 36% (32/90) of patients [12]. On quantitative flow assessment, compared with 90 age-, sex- and comorbidity-matched controls (studied pre-COVID-19), there was no significant difference in global hyperaemic MBF. The authors concluded that the high prevalence of regional ischaemia and/or infarction (40%) suggested the likely presence of pre-existing occult CAD, reflecting the demographics and comorbidity burden of the population studied (elderly with multiple cardiovascular risk factors).

Limitations inherent in previous studies include selection bias from the study of patients referred clinically for ischaemia assessment and the use of convenience samples of patients presenting with chest pain or dyspnoea (some studies excluding those with prior CAD). The majority have also been retrospective analyses, with a wide interval between the index admission and perfusion assessment. Another limitation is the lack of appropriate contemporaneous, matched controls. Nonetheless, these data are largely consistent with our findings, taken from a prospective study with matched controls, that myocardial ischaemia is not significantly more prevalent than in a matched control population.

Subjects studied in our analysis were imaged a median of 21 days after hospital discharge. Although this is earlier than in all previously published studies, it is still possible that pro-inflammatory changes and hypercoagulability may be transient phenomena which may have resolved either spontaneously or with medical therapy (with antiplatelet/anticoagulant agents) prior to CMR assessment. Hence, the pathophysiological changes responsible for myocardial damage may not have been captured at the time of CMR assessment, and the extent of hypoperfusion in the acute phase, underestimated. There is some evidence that microvascular dysfunction in COVID-19 may be regional, in contrast to the global microvascular dysfunction that is observed in non-obstructive CAD or in association with type 2 diabetes. This may reflect pathophysiological differences in the structural and functional elements of disease, with transient thrombosis and inflammation being more prominent in COVID-19.

An interesting observation is that a significant proportion of patients with myocardial injury did not have stress perfusion defects. A number of explanations may apply. Firstly, stress perfusion imaging may lack sensitivity relative to LGE imaging for myocardial injury, owing to its lower spatial resolution and its imaging being confined to three short-axis slices (compared with full left ventricular coverage with LGE imaging). Secondly, microvascular abnormalities may also be regional, corresponding to the microinfarcts observed in 9% of cases. Even quantitative flow assessment may lack sensitivity in identifying these regional differences, though a pixel-wise approach, as used in the majority of our participants, is a step forward. Thirdly, it is possible that troponin elevation may relate not only to macro- or microvascular disease mechanisms, but also to myocardial inflammation/myocarditis including direct viral cytopathic effects, or type 2 myocardial infarction (due to oxygen supply–demand imbalance). Another potential injury mechanism in COVID-19, which may underlie discordance between scarring and hypoperfusion, is the occurrence of tissue ischaemia with normal [or even high] myocardial blood flow. However, the observed patterns of injury (namely predominantly macro and/or microinfarcts) indicate that damage may be mediated by vascular mechanisms, though this may be due to a transient prothrombotic state.

### Study limitations

A limitation of our study is that only a subset of participants in COVID-HEART underwent stress perfusion imaging, with the latter being determined according to the availability of stress perfusion CMR in the recruiting centre and physician/patient choice. Other potential sources of bias include the exclusion of patients with contraindications to CMR and the exclusive recruitment of subjects with biochemical evidence of myocardial injury: hence, the results of this work may not be representative of wider COVID-19 population (namely those without overt cardiac injury, and non-hospitalised patients, in whom ischaemia may still occur). Nonetheless, this remains a real-world multicentre clinical cohort recruited prospectively and compared with contemporaneous controls analysed in a blinded core lab. However, survivor bias remains another possible limitation: more severe injury and microvascular obstruction may occur in more severe/end-stage disease (as indicated in some autopsy series). Without anatomical assessment (invasive angiography or computed tomography coronary angiography), the prevalence of epicardial CAD could not be determined, and without invasive assessment, CMR-determined microvascular dysfunction could not be definitively confirmed as such (as opposed to multivessel CAD).

## Conclusions

In this predefined analysis of a prospective observational study evaluating patients recently hospitalised with COVID-19 and with serum troponin elevation, the frequency of persistent myocardial ischaemia was relatively low and not significantly different from matched controls. This finding coupled with the lack of an association between ischaemia and myocardial scar suggests that epicardial coronary abnormalities may not be the predominant mechanism underlying COVID-induced myocardial injury.

## Supplementary Information

Below is the link to the electronic supplementary material.

Supplementary file1 (DOCX 16 KB)

Supplementary file1 (DOCX 15 KB)

## Data Availability

No datasets were generated or analysed during the current study.
