# Supplementary material for: Myocardial ischaemia following COVID-19: a cardiovascular magnetic resonance study
Source: Int J Cardiovasc Imaging. 2024 Dec 30;41(2):247–56. doi: 10.1007/s10554-024-03304-7 (PMC11811239; doi:10.1007/s10554-024-03304-7)
Supplement: Supplementary file 1 — Supplementary file1 (DOCX 16 KB) [file 10554_2024_3304_MOESM1_ESM.docx]

**Supplementary table 1: Clinical characteristics of COVID+/troponin+ patients with and without CMR perfusion assessment.**

|  | **CMR perfusion imaging available (n=59)** | **CMR perfusion imaging not available (n=283)** | **All** | **P-value** |
| --- | --- | --- | --- | --- |
| Age (years) | 60.8±11.4 | 60.0±12.5 | 60.0±12.5 | 0.651 |
| Sex (Male) | 49 (83.1%) | 194 (68.6%) | 243 (71.1%) | 0.025 |
| Body Mass Index (kg/m2) | 29.9±6.3 | 29.9±6.6 | 29.9±6.5 | 0.977 |
| Charlson Score | 2.64±1.89 | 2.54±1.91 | 2.56±1.91 | 0.726 |
| Haemoglobin (g/L) | 137±20.7 | 137.1±18.6 | 137.05±18.9 | 0.982 |
| eGFR (ml/min/1.73m2) | 63.2±22.6 | 68.4±19.5 | 67.6±20.1 | 0.094 |
| Time of CMR post-hospital discharge (days) | 21 [15-27] | 20 [9-28] | 21 [11.5-27] | 0.575 |
| Hospital length of stay (days) | 7 [4-13] | 9 [5-18] | 9 [5-16] | 0.130 |
| Ethnicity (White) | 30 (50.8%) | 196 (69.2%) | 226 (66.1%) | 0.007 |
| MI/ACS | 8 (13.6%) | 26 (9.2%) | 34 (9.9%) | 0.307 |
| Previous Revascularisation | 9 (15.5%) | 22 (7.5%) | 31 (9.1%) | 0.069 |
| Hypertension | 27 (45.8%) | 133 (47.0%) | 160 (46.8%) | 0.863 |
| Heart failure | 6 (10.2%) | 16 (5.7%) | 22 (6.4%) | 0.238 |
| Smoking Status |  |  |  | 0.060 |
| Current | 0 (0%) | 21 (7.4%) | 21 (6.4%) |  |
| Former | 18 (30.5%) | 88 (31.1%) | 106 (31.0%) |  |
| Never | 41 (69.5%) | 174 (61.5%) | 215 (62.9%) |  |
| Diabetes (type 1) | 0 (0%) | 6 (2.1%) | 6 (1.8%) | 0.595 |
| Diabetes (type 2) | 13 (22.0%) | 65 (30.0%) | 78 (22.8%) | 0.876 |
| Diabetes (Any) | 13 (22.0%) | 71 (25.1%) | 84 (24.6%) | 0.620 |
| Aspirin/Clopidogrel/Ticagrelor | 15 (25.4%) | 60 (21.2%) | 75 (21.9%) | 0.476 |
| Statin | 27 (45.8%) | 119 (42.0%) | 146 (42.7%) | 0.600 |
| Beta-blocker | 20 (33.9%) | 58 (20.5%) | 78 (22.8%) | 0.026 |
| ACE-I or ARB | 28 (47.5%) | 93 (32.9%) | 121 (35.4%) | 0.033 |
| Anti-coagulant | 4 (6.8%) | 18 (6.4%) | 22 (6.4%) | 1 |

Data presented are mean ± SD, median [Q1-Q3], or n (%). Abbreviations: *ACE-I* angiotensin converting enzyme inhibitor; *ARB* angiotensin receptor blocker; *ACS* acute coronary syndrome; *CMR* cardiovascular magnetic resonance imaging; *MI* myocardial infarction.

**Supplementary table 2: Imaging characteristics of COVID-19 patients with and without CMR perfusion assessment.**

|  | **CMR perfusion imaging available (n=59)** | **CMR**  **perfusion imaging**  **not available**  **(n=283)** | **All** | **P-value** |
| --- | --- | --- | --- | --- |
| Left ventricular end-diastolic volume index (ml/m^2^) | 83.2±27.6 | 78.6±26.2 | 79.5±26.5 | 0.210 |
| Left ventricular end-systolic volume index (ml/m^2^) | 33.4±27.8 | 31.0±21.1 | 31.4±22.4 | 0.536 |
| Left ventricular mass index (g/m^2^) | 62.5±18.1 | 58.1±16.2 | 58.8±16.6 | 0.060 |
| Left ventricular ejection fraction (%) | 62.9±13.6 | 62.6±12.0 | 62.6±12.3 | 0.824 |
| LVMi/LVEDVi (g/ml) | 0.78±0.21 | 0.77±0.22 | 0.77±0.22 | 0.863 |
| LGE present | 27 (45.8%) | 108 (40.6%) | 135 (42%) | 0.467 |

Data presented are mean ± SD, median [Q1-Q3], or n (%). Abbreviations: *CMR* cardiovascular magnetic resonance imaging; *LGE* late gadolinium enhancement. *LVMi* left ventricular end-diastolic mass indexed to body surface area; *LVEDVi* left ventricular end-diastolic volume indexed to body surface area. LGE images analysable; n=325).
