# Supplementary material for: Myocardial ischaemia following COVID-19: a cardiovascular magnetic resonance study
Source: Int J Cardiovasc Imaging. 2024 Dec 30;41(2):247–56. doi: 10.1007/s10554-024-03304-7 (PMC11811239; doi:10.1007/s10554-024-03304-7)
Supplement: Supplementary file 2 — Supplementary file1 (DOCX 15 KB) [file 10554_2024_3304_MOESM2_ESM.docx]

**Appendix**

List of investigators Chief Investigator: JP Greenwood; Grant applicants: JP Greenwood, GP McCann, C Berry, M Dweck, JC Moon, CM Miller, A Chiribiri, S Prasad, VM Ferreira, C Bucciarelli-Ducci, D Dawson; Trial Analysis Group: James C. Moon (chair), John P. Greenwood, Jessica Artico, Hunain Shiwani, Rhodri Davies, Vanessa M. Ferreira, Marc Dweck, Colin Berry, Giles Roditi, , Robin Young, Alex McConnachie, Bernard Kelly, Peter W. Macfarlane, Gerry P. McCann, Christopher A. Miller; Trial sites: Leeds Teaching Hospitals NHS Trust: John P. Greenwood, Eylem Levelt, Miroslawa Goreka, Kathryn Somers, Roo J. Byrom-Goulthorp, Michelle Anderson, Laura Britton, Fiona Richards, Laura M. Jones; University Hospitals of Leicester NHS Trust: Gerry P. McCann, Ranjit Arnold, Alastair Moss, Jude Fisher, Joanne Wormleighton, Kelly Parke, Rachel England, Jian Yeo; NHS Grampian: Dana Dawson, Judith Falconer, Valerie Harries, Paula Henderson; NHS Lothian: Marc Dweck, Trisha Singh, David Newby; Oxford University Hospitals NHS Foundation Trust: Vanessa M. Ferreira, Stefan K Piechnik, Iulia Popescu, Elena Lukaschuk, Qiang Zhang, Mayooran Shanmuganathan, Stefan Neubauer, Betty Raman, Keith Channon, Catherine Krasopoulos, Claudia Nunes, Liliana Da Silva Rodrigues, Harriet Nixon, Athanasia Panopoulou, Alison Fletcher, Peter Manley; NHS Greater Glasgow and Clyde: Colin Berry, Kenneth Mangion, Andrew Morrow, Robert Sykes, Kirsty Fallon, Ammani Brown, Laura Kelly, Christopher McGinley, Michael Briscoe, Rosemary Woodward, Tracey Hopkins, Evonne McLennan, Nicola Tynan, Laura Dymock; Mid Yorkshire Hospitals NHS Trust: Peter Swoboda, Judith Wright, Donna Exley; Birmingham NHS Foundation Trust: Richard Steeds, Kady Hutton, Sonia MacDonald; University College London Hospitals NHS Foundation Trust: James C. Moon, Thomas Treibel, Jessica Artico, Abhishek Shetye; Manchester University NHS Foundation Trust: Christopher M. Miller, Christopher Orsborne, William Woodville-Jones, Susan Ferguson, Konstantinos Bratis; Liverpool Heart and Chest Hospital NHS Foundation Trust: Timothy Fairbairn, Michail Sionas, Peris Widdows, Pei Gee Chew, Christian Marsden, Tom Collins, Linsha George, Lisa Kearney; University Hospital Southampton NHS Foundation Trust: Andrew Flett, Simon Smith, Alice Zhenge, Jake Harvey, Liliana Inacio, Tomas Hanam-Penfold, Lucy Gruner; Royal Free London NHS Foundation Trust: Marianna Fontana, Yousuf S.K. Razvi, Jacolene Crause, Nina M. Davies, Jessica Artico, James T. Brown, Liza Chaco, Rishi Patel, Tushar Kotecha, Dan S. Knight; Northumbria Healthcare NHS Foundation Trust: Thomas Green, David Ripley, Maria Thompson; Guy’s and St Thomas NHS Foundation Trust: Amedeo Chiribiri, Ugochi Akerele, Elna Cifra, Ebraham Alskaf, Richard Crawley, Adriana Villa; University Hospitals Bristol NHS Foundation Trust: Chiara Bucciarelli-Ducci, Angus K. Nightingale, Kim Wright, Esther D. Bonnick, Emma Hopkins, Jessy George, Linta Joseph; Imperial College Healthcare NHS Trust: Graham Cole, Kavitha Vimalesvaran, Nadine Ali, Caitlin R. Carr, Alexandra A.R. Ross, Clara King; Royal Brompton and Harefield NHS Foundation Trust: Sanjay Prasad, Zohreh Farzad, Sara A. Salmi, Kevin Kirby; Newcastle Upon Tyne Hospitals NHS Foundation Trust: Adam McDiarmid, Hannah J. Stevenson, Pamela S. Matsvimbo, Lency Joji, Margaret Fearby, Benjamin Brown; St George's University Hospitals NHS Foundation Trust: Nicholas Bunce, Robert Jennings, Vennessa Sookhoo, Shatabdi Joshi; Liverpool University Hospitals NHS Foundation Trust: Prathap Kanagala, Sandra Fullalove, Catherine Toohey, Kate Fenlon; The Royal Devon and Exeter Hospital Foundation Trust: Nicholas Bellenger, Jingzhou He, Sarah Statton, Nicola Pamphilon, Anna Steele, Claire Ball, Ann McGahey, Silvia Balma, Lynsey Wilkes, Katy Lewis, Michelle Walter; Swansea Bay University Health Board: Adrian Ionescu, Tishi Ninan, Suzanne Richards, Marie Williams; Lewisham and Greenwich NHS Trust: Khaled Alfakih, Samia Pilgrim; Barts Health NHS Trust: James C. Moon, Jessica Artico, George Joy, Charlotte H. Manisty, Ifza Hussain, Thomas Treibel.
